# The Role of Ventricular Assist Devices in Patients With Heart Failure Due to Dilated Cardiomyopathy: A Systematic Review

**DOI:** 10.7759/cureus.66259

**Published:** 2024-08-06

**Authors:** Billy McBenedict, Wilhelmina N Hauwanga, Emmanuel S Amadi, Melvin Chun Yang Yau, Chibuike R Amuzie, Mujahid O Abdelraof, Berley Alphonse, Abdullah Mohammed Abdullah Ibrahim, Madeleine O Okere, Ogbonna Chikere, Chukwuwike Johnny, Bruno Lima Pessôa

**Affiliations:** 1 Neurosurgery, Fluminense Federal University, Niterói, BRA; 2 Family Medicine, Faculty of Medicine, Federal University of the State of Rio de Janeiro, Rio de Janeiro, BRA; 3 Internal Medicine, Hallel Hospital, Port Harcourt, NGA; 4 Medicine, Monash University Malaysia, Subang Jaya, MYS; 5 Public Health, Louisiana State University Shreveport, Shreveport, USA; 6 Medicine and Surgery, Alzaiem Alazhari University, Khartoum, SDN; 7 Internal Medicine, University Notre Dame of Haiti, Port-au-Prince, HTI; 8 Internal Medicine, Faculty of Medicine, University of Gezira, Wad Madani, SDN; 9 Internal Medicine, University of Port Harcourt Teaching Hospital, Port Harcourt, NGA; 10 Internal Medicine, St. Luke's Hospital, St. Louis, USA

**Keywords:** cardiac transplantation, mechanical circulatory support, heart failure management, ventricular assist devices, dilated cardiomyopathy

## Abstract

Dilated cardiomyopathy (DCM) is a prevalent heart muscle disease characterized by ventricular dilation and systolic dysfunction, leading to severe heart failure (HF) and often requiring heart transplantation (HTx). This systematic review aimed to synthesize information regarding the role of ventricular assist devices (VADs) in managing HF patients due to DCM. A comprehensive search was conducted across PubMed, Embase, Scopus, Web of Science, and Cochrane databases for studies published between 2014 and 2024. Inclusion criteria were studies involving adult patients with HF due to DCM treated with VADs. Exclusion criteria included non-human studies, pediatric populations, and non-peer-reviewed articles. Thirty-one studies met the inclusion criteria. The included studies demonstrated that the use of VADs in patients with DCM resulted in significant improvements in left ventricular ejection fraction (LVEF), myocardial fibrosis reduction, and reverse ventricular remodeling. Studies reported enhanced survival rates, reduced symptoms, and better quality of life. VADs served as a critical bridge to HTx and, in some cases, as long-term destination therapy. However, complications such as thrombus formation, anemia, and kidney failure were noted, emphasizing the need for vigilant monitoring and management. Continuous advancements in VAD technology and patient management protocols were found to be essential for optimizing outcomes. We conclude that VADs play a crucial role in managing advanced HF due to DCM by providing mechanical circulatory support, improving cardiac function, and enhancing patient survival and quality of life. Despite associated complications, VADs are invaluable for patients with severe HF, offering both immediate and long-term therapeutic benefits. Future research should focus on minimizing complications and further improving VAD technology to enhance patient outcomes.

## Introduction and background

Dilated cardiomyopathy (DCM) is a heart muscle disease characterized by left ventricular (LV) or biventricular dilation and systolic dysfunction in the absence of either pressure or volume overload or coronary artery disease (CAD) sufficient enough to explain the dysfunction [1]. DCM is marked by the dilation and impaired contraction of the ventricles, primarily affecting the left ventricle and resulting in systolic dysfunction. This condition increases ventricular volumes to maintain cardiac output, leading to the thin-walled, dilated appearance of the left ventricle. Genetic mutations are significant contributors to DCM, impacting various intracellular structures and pathways. Key mechanisms include deficits in force generation due to mutations in sarcomeric proteins like titin and myosin, defects in the nuclear envelope involving Lamin-A/C mutations, and issues with force transmission linked to cytoskeletal protein mutations such as filamins and dystrophin. Additionally, abnormalities in cell-to-cell adhesion from desmosomal protein mutations, mitochondrial energy production defects, calcium-cycling issues from phospholamban gene mutations, ion channel mutations, epigenetic perturbations, and protein misfolding diseases all contribute to the pathophysiology of DCM [1].

Cardiac remodeling in DCM involves significant alterations in function, particularly in the LV pressure-volume relationship. Increased end-diastolic volumes and pressures, along with diastolic dysfunction due to incomplete relaxation and increased stiffness, complicate the clinical scenario [1]. The law of Laplace explains that wall tension is directly proportional to ventricular dilation and inversely proportional to wall thickness, highlighting the increased afterload and energetic consequences of heart failure (HF) [1]. Understanding these genetic and molecular mechanisms is crucial for developing targeted therapies and improving outcomes for DCM patients [2].

Histological examination of the myocardium typically shows nonspecific changes of fibrosis and hypertrophy, along with myocardial injury marked by an inflammatory cell infiltrate [2]. It is the most common form of cardiomyopathy and the most frequent indication for cardiac transplantation. The incidence of DCM in adults is about 5.5 per 100,000 people per year, while the incidence in children is between 0.34 and 1.09 per 100,000 people per year [3,4]. Affected individuals may present with a variety of symptoms, most commonly those associated with HF, such as progressive dyspnea with exertion, impaired exercise capacity, orthopnea, paroxysmal nocturnal dyspnea, and peripheral edema. Other presentations include the incidental detection of asymptomatic cardiomegaly and symptoms related to coexisting arrhythmias, conduction disturbances, thromboembolic complications, or sudden death [5,6]. DCM is the third leading cause of HF and the most common indication for heart transplantation (HTx) [7]. It is also a common cause of sudden cardiac death in young people.

DCM can be classified as either primary (idiopathic/genetic) or secondary (acquired factors). In idiopathic cases, up to 50% of patients show no identifiable cause even after extensive evaluations [8]. Approximately 35% of DCM cases have a hereditary or genetic basis, with mutations typically affecting genes related to cytoskeletal, sarcomere, and nuclear envelope proteins [9]. Secondary causes of DCM include infectious myocarditis (e.g., viral, Chagas disease, Lyme disease), ischemic disease, hypertension, medication-induced cardiotoxicity (e.g., anthracyclines), alcohol abuse, HIV, peripartum cardiomyopathy, and infiltrative diseases. Ischemic cardiomyopathy due to CAD is the leading cause of congestive HF but is considered distinct from DCM unless CAD is occult. Stress cardiomyopathy, also known as Takotsubo cardiomyopathy or Broken Heart Syndrome, is a relatively rare but increasingly recognized cause, typically classified separately from primary DCM [10].

The prognosis for DCM depends on the severity of the disease and the potential for reversing heart changes. Patients with the lowest ejection fractions or significant diastolic dysfunction have the poorest outcomes, often progressing to terminal HF, requiring an LV assist device or HTx [9]. As the disease advances, patients often experience a decline in quality of life due to severe symptoms, frequent hospitalizations, and the psychological burden of living with a chronic, potentially fatal illness.

DCM is more commonly observed in men than in women, with an estimated prevalence of 36 cases per 100,000 in the general population. In the United States, DCM accounts for approximately 10,000 deaths and 46,000 hospitalizations annually. However, these figures may underestimate the true prevalence because many patients are asymptomatic and thus undiagnosed despite having LV dysfunction [11,12]. The treatment for DCM includes medical therapy with beta-blockers, angiotensin-converting enzyme inhibitors or angiotensin receptor blockers, aldosterone antagonists, diuretics, and anticoagulants to manage HF symptoms and prevent complications. Device therapy, such as implantable cardioverter defibrillators, cardiac resynchronization therapy, and ventricular assist devices (VADs), supports heart function and prevents sudden cardiac death. Lifestyle modifications like a low-sodium diet, regular exercise, weight management, and avoiding alcohol and smoking are crucial.

VADs are mechanical pumps that enhance cardiac function and improve blood flow in patients with weakened ventricles. These devices can take over some or all of the heart's pumping duties, ensuring adequate circulation throughout the body. VADs are essential for supporting individuals with severe HF by providing critical assistance when the heart's pumping ability is compromised. Studies have shown the effectiveness of VADs in patients with DCM in both pediatric and adult populations. For instance, a cohort study on adults reported satisfactory survival rates using the latest generation Abbott HeartMate 3 LVAD for long-term mechanical circulatory support, either as a bridge to transplantation or destination therapy [13]. Similarly, a pediatric cohort study found that patients with DCM were the largest subset and exhibited the best survival rates [14]. These findings highlight the benefits of VADs in improving survival rates and overall health outcomes. This systematic review aims to explore the role of VADs in managing HF due to DCM, focusing on their clinical effectiveness, patient outcomes, and comparative benefits over other treatments.

## Review

Materials and methods

The systematic review adhered to the principles outlined in the Preferred Reporting Items for Systematic Reviews and Meta-Analyses (PRISMA) guidelines for the organization and reporting of its results [15]. An electronic search was performed across multiple research databases, including PubMed, Embase, Scopus, Web of Science, and Cochrane (Table 1). All databases were accessed on May 16, 2024, and subsequently, a search was performed.

**Table 1:** Summary of the search strategy from the various databases

Database	Search strategy	Filters used
PubMed	("outcomes"[Title/Abstract] OR "results"[Title/Abstract]) AND ("Ventricular Assist Devices"[Title/Abstract] OR "VAD"[Title/Abstract] OR "LVAD"[Title/Abstract] OR "RVAD") AND ("Dilated Cardiomyopathy"[Title/Abstract])	Humans only, English language, exclude preprints, filter years 2014-2024
Embase	('outcomes':ab,ti OR 'results':ab,ti) AND ('ventricular assist devices':ab,ti OR 'vad':ab,ti OR 'lvad':ab,ti OR 'rvad':ab,ti) AND 'dilated cardiomyopathy':ab,ti)	Humans only, English language, filter years 2014-2024
Scopus	TITLE-ABS-KEY ("outcomes" OR "results") AND ("Ventricular Assist Devices" OR "VAD" OR "LVAD" OR "RVAD") AND ("Dilated Cardiomyopathy")	Humans only, English language, filter years 2014-2024
Web of Science	((AB=("Trigeminal Neuralgia" OR "Trifacial Neuralgia" OR "Tic Douloureux")) AND AB=("Surgical Interventions" OR "Surgery" OR "Operative Procedures")) AND AB=("Outcomes" OR "Results" OR "Efficacy")	Humans only, English language, filter years 2014-2024
Cochrane	#1 ("outcomes" OR "results"):ti,ab,kw (Word variations have been searched) #2 ("Dilated Cardiomyopathy"):ti,ab,kw (Word variations have been searched) #3 "Ventricular Assist Devices" OR "VAD" OR "LVAD" OR "RVAD" #4 (#1 AND #2 AND #3)	Humans only, English language, filter years 2014-2024

Inclusion and Exclusion Criteria

The inclusion criteria encompassed studies involving human subjects (adults) diagnosed with HF due to DCM who were treated with ventricular assist devices (VADs) and had patient follow-up data. Eligible study designs included primary research studies published in English. Studies of interest reported on patient follow-up for individuals with HF due to DCM treated with VADs. Only peer-reviewed journal articles in English were considered for inclusion. Exclusion criteria included non-human, pediatric population, or studies that did not directly provide information on patient follow-up for HF due to DCM with VADs, non-peer-reviewed articles, conference abstracts, opinion pieces, and editorials.

Results

**Figure 1:**
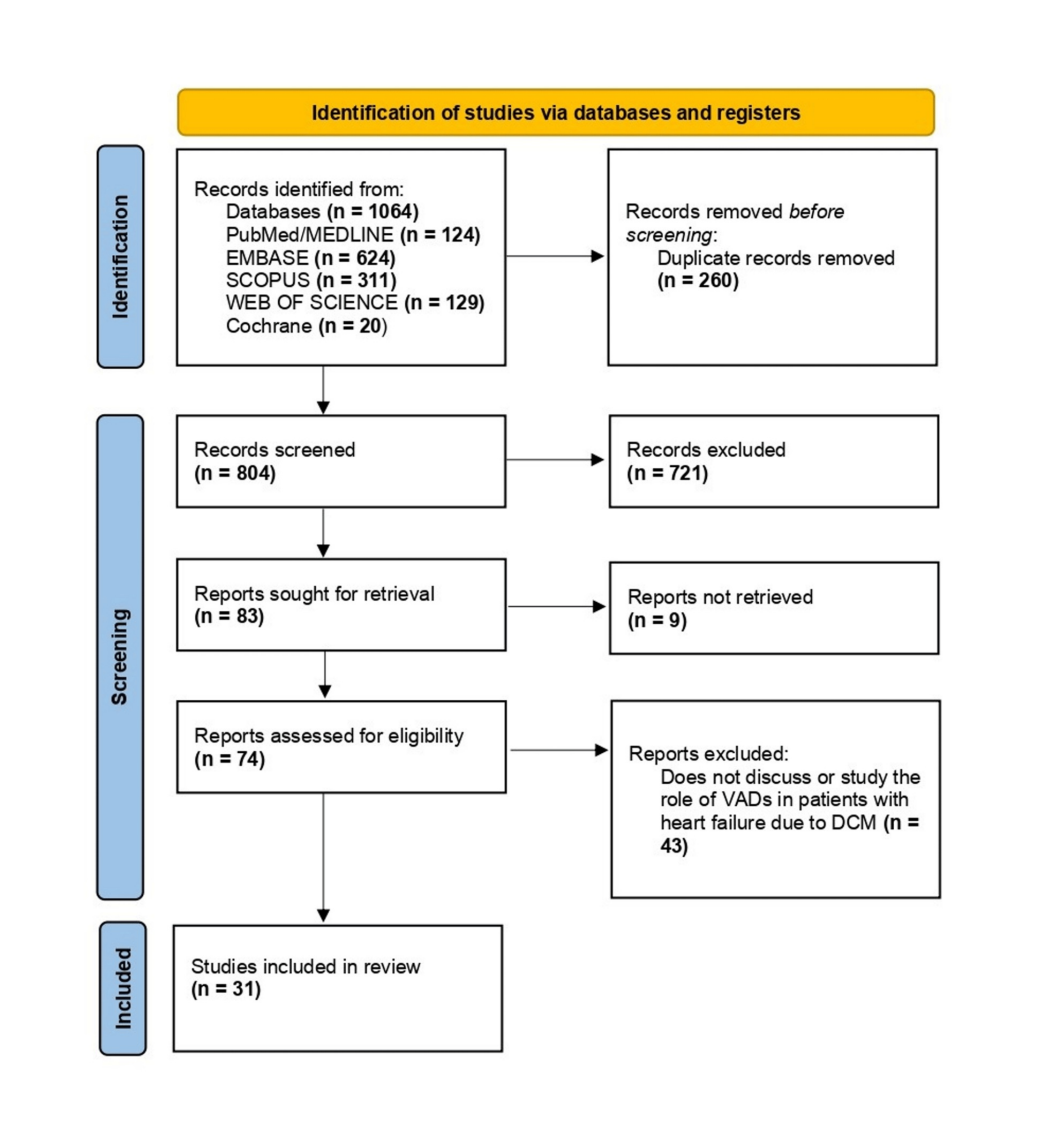
Preferred Reporting Items for Systematic Reviews and Meta-Analyses (PRISMA) flow diagram indicating the steps taken to filter the articles for this review

Through our search strategy, we identified a total of 1064 articles (Figure 1), including 124 from PubMed/Medline, 624 from Embase, 311 from Scopus, 129 from Web of Science, and 20 from Cochrane. We applied filters based on inclusion and exclusion criteria and then transferred the articles to an Excel sheet. After manually removing 260 duplicates, 804 articles remained. These were further scrutinized based on titles and abstracts, leading to the exclusion of 721 articles, leaving 83 for further consideration. We were unable to retrieve the full texts for 9 articles, resulting in 74 papers eligible for assessment. Following a thorough full-text review, 43 papers were excluded, culminating in 31 articles included in the final review (Table 2). Data screening was independently conducted by two review authors, with a third reviewer consulted in cases of disagreement. Importantly, no automated tools were utilized in this process.

**Table 2:** Studies that were used to synthesize this review, with their respective demographics and key results VADs: ventricular assist devices; HF: heart failure; DCM: dilated cardiomyopathy; VA: ventricular arrhythmias; LVAD: left ventricular assist devices; ICD: implantable cardioverter defibrillator; LVEF: left ventricular ejection fraction; FDCM: familial dilated cardiomyopathy; HTx: heart transplantation; HVADs: HeartWare ventricular assist devices; NIDCM: non-ischemic dilated cardiomyopathy; IDCM: idiopathic dilated cardiomyopathy; BNP: brain natriuretic peptide; PF: pulsatile flow; LVRR: left ventricular reverse remodeling; ICM: ischemic cardiomyopathy

Author	Demographic data	Key findings
Oishi et al. [16]	11 patients: 9 men (81.8%) and 2 women (18.2%)	The Impella 5.0 VADs effectively support patients with advanced HF, including those with DCM, though prolonged use increases the risk of purge system malfunctions, necessitating careful monitoring and strategic management.
Darma et al. [17]	357 patients: 314 men (88%) and 43 women (12%); control group: 193 men (85%) and 34 women (15%); late VA group: 121 men (93%) and 9 women (7%)	VADs significantly improve survival rates and cardiac function in patients with HF due to DCM, facilitating substantial myocardial recovery and enhancing overall patient outcomes.
Zormpas et al. [18]	253 patients: 216 men (85%) and 37 women (15%)	LVAD implantation significantly improves prognosis, HF symptoms, and survival rates in patients with DCM, facilitating myocardial recovery and serving as both a bridge to transplant and long-term therapy.
Schlöglhofer et al. [19]	24 patients: 19 men (79.2%) and 5 women (20.8%)	The HeartMate 3 LVAD significantly improves outcomes in patients with HF due to DCM by providing effective mechanical support and enhanced hemodynamic stability through advanced monitoring and optimization techniques.
Wasilewski et al. [20]	78 patients: 72 men (92%) and 6 women (8%)	VADs significantly improve survival and quality of life in patients with severe HF due to DCM, serving as a crucial intervention for those who have exhausted conventional medical therapies.
Wasilewski et al. [21]	125 patients with LVAD Implantation. 90 patients with full 12 months of follow-up: 85 men (94%) and 5 women (6%)	LVADs significantly improve survival and quality of life in patients with HF due to DCM by providing mechanical support that enhances hemodynamic stability and organ perfusion, despite complications such as kidney failure and anemia.
Hofmeyer et al. [22]	1198 patients: 674 men (56.3%) and 524 women (43.7%) 347 patients with LVAD: 227 men (65.4%) and 120 women (34.6%) 511 patients with ICD: 274 men (53.6%) and 237 women (46.4%) 340 patients with neither LVAD nor ICD: 173 men (50.9%) and 167 women (49.1%)	VADs are crucial for managing DCM, particularly in patients with severe genetic markers, serving as both a bridge to transplantation and a destination therapy.
Selzman et al. [23]	150 patients: 119 men (79%) and 31 women (21%)	The Jarvik 2000 LVAD significantly supports patients with end-stage HF as a bridge to transplantation, with a success rate of 67.3%, improving to 91% with the updated cone bearings, which reduced complications such as hemolysis and organ dysfunction.
Gyoten et al. [24]	12 patients: 10 men (83%) and 2 women (17%)	VADs significantly improve cardiac function and facilitate myocardial recovery in patients with DCM, with a substantial increase in LVEF.
Antonides et al. [25]	28 patients with follow-up: 23 men (82.1%) and 5 women (17.9%)	LVADs are effective in facilitating myocardial recovery and improving long-term survival rates in patients with severe HF due to DCM, enabling successful device explantation and significant improvements in cardiac function and quality of life.
Vela et al. [26]	26 patients: 22 men (84%) and 4 women (16%)	LVADs significantly support patients with severe HF due to idiopathic DCM, leading to substantial myocardial recovery and high survival rates post-explantation.
Efimova et al. [27]	98 patients with LVAD Implantation: 86 men (87.76%) and 12 women (12.24%)	LVADs significantly improve survival rates and facilitate myocardial recovery in patients with severe HF due to DCM, despite the high incidence of complications such as VAs.
Khayata et al. [28]	24,809 patients: 13,769 men (84%) and 11,040 women (44.5%)	VADs, though less frequently used in patients with FDCM, are crucial for stabilizing these patients and bridging them to HTx.
William et al. [29]	75 patients: 66 men (88%) and 9 women (12%)	VADs significantly improve myocardial recovery and clinical outcomes in patients with DCM by effectively unloading the left ventricle, with optimal mechanical unloading leading to better heart function and reduced complications.
Shehab et al. [30]	13 patients: 10 men (76,92%) and 3 women (23,08%)	Dual HVADs effectively support patients with severe biventricular failure due to DCM, significantly improving survival rates and enabling successful bridging to HTx, despite a high rate of complications.
Sugimura et al. [31]	50 patients: 42 men (84%) and 8 women (16%)	VADs, specifically the Impella 5+, are crucial for managing acute HF in patients with DCM, improving cardiac output, and stabilizing systemic blood pressure, though patients with biventricular failure may exhibit higher in-hospital mortality.
Panoulas et al. [32]	92 patients: 72 men (78.3%) and 20 women (21.7%)	VADs, particularly the Impella, effectively support patients with DCM by bridging them to recovery, durable LVAD implantation, or HTx, with an estimated one-year survival rate of 80% for those transitioning to definitive treatments.
Koga-Ikuta et al. [33]	50 patients: 41 men (82%) and 9 women (18%)	VADs significantly contribute to functional recovery in patients with NIDCM by providing mechanical support and facilitating myocardial remodeling, particularly in those with higher numbers of immunocompetent cells in the myocardium.
Ito et al. [34]	14 patients: 10 men (71.4%) and 4 women (28.6%)	LVADs significantly improve cardiac structure and function in patients with end-stage nonischemic DCM by inducing beneficial histopathological and epigenetic modifications, including changes in histone methylation.
Glass et al. [35]	22 patients with HVAD Implants: 19 men (86%) and 3 women (14%)	The HVAD, while effective in providing support for patients with HF including DCM, has a high incidence of thrombus formation, particularly on the inflow cannula, leading to significant thromboembolic events despite standard anti-coagulation protocols.
Sammani et al. [36]	489 patients: 372 men (76%) and 117 women (24%)	LVADs significantly extended the lives of patients with severe HF due to DCM, serving as both a bridge to HTx and, in some cases, as destination therapy, thereby improving quality of life while awaiting a donor's heart.
Cherbi et al. [37]	772 patients: 552 men (71.5%) and 220 women (28.5%)	VADs play a crucial role in managing advanced IDCM, especially in patients under 65, significantly increasing the likelihood of requiring VADs or HTx due to the severe progression of IDCM.
Yagi et al. [38]	120 patients: 89 men (74.2%) and 31 women (25.8%)	LVADs significantly improve clinical outcomes and hemodynamic stability in patients with DCM, reducing BNP levels and serving effectively as a bridge to transplant or recovery.
Imamura et al. [39]	60 patients: 48 men (80%) and 12 women (20%)	PF LVADs significantly facilitate LVRR in patients with stage D HF due to DCM, enhancing myocardial recovery and improving cardiac function, particularly when combined with adequate preoperative beta-blocker treatment.
Ivanov et al. [40]	36 patients ICM: 33 men (92%) and 3 women (8%) 24 patients DCM: 21 men (87%) and 3 women (13%)	VADs effectively manage end-stage HF in patients with both ICM and DCM, providing comparable survival benefits and serving as a critical bridge to transplantation or destination therapy without significant differences in survival outcomes between the two groups.
Manca et al. [41]	800 patients: 569 men (71.1%) and 231 women (28.9%)	VADs are crucial for patients with non-ischemic DCM who experience transient rather than sustained improvements in LVEF, as these patients have a higher risk of adverse outcomes and more frequently require VADs.
Broch et al. [42]	102 patients: 74 men (73%) and 28 women (27%)	While LVADs were used selectively as a bridge to transplantation in patients with HF due to DCM, they were crucial for those whose condition did not sufficiently improve with pharmacological treatment alone, highlighting better outcomes with early intervention in severe cases.
Kuśmierczyk et al. [43]	38 patients: 32 men (84.2%) and 6 women (15.8%)	LVADs significantly improve survival and quality of life in patients with severe HF not eligible for transplantation, with continuous-flow LVADs showing a notably higher annual survival rate (82%) compared to PF devices (61%).
Biełka et al. [44]	79 patients: 77 men (97.5%) and 2 women (2.5%)	Continuous-flow LVADs are crucial in supporting patients with HF due to DCM, with NICM patients showing higher rates of recovery and device explantation compared to ICM patients, who experienced more gastrointestinal bleeding despite similar long-term survival rates.
Parikh et al. [45]	8 patients with LVAD (VAD group): 7 men (87.5%) and 1 woman (12.5%) 8 patients without LVAD (NVAD group): 7 men (87.5%) and 1 woman (12.5%)	VADs not only provide mechanical support but also induce significant favorable gene expression changes in both the left and right ventricles of patients with DCM, contributing to reverse remodeling and potentially aiding in heart function recovery.
Frazier et al. [46]	27 patients (explant group): 16 men (59.26%) and 11 women (40.74%)	LVADs significantly improve cardiac function in patients with HF due to DCM, facilitating the explantation process and enabling patients to maintain improved heart function and survival post-explantation.

Study Quality and Bias Assessment

The Joanna Briggs Institute (JBI) Critical Appraisal Checklist identifies several common challenges that can affect the validity and reliability of articles included in the systematic review. These challenges include selection bias due to non-comparable groups, measurement bias from invalid or unreliable exposure and outcome assessments, and confounding from unidentified or inadequately controlled confounding factors. Additional issues include attrition bias from loss to follow-up, inappropriate statistical analysis, and reporting bias due to selective or non-transparent reporting of results. Addressing these challenges is crucial to ensure accurate and reliable findings in cohort studies, enabling their application in clinical practice and further research. The quality of the 31 articles was assessed using the JBI Critical Appraisal Tools (see Table 3 in the Appendix). All studies included in the analysis focused on a clearly defined issue regarding the role of VADs in patients with HF due to DCM. Each study recruited participants in an acceptable manner, clearly stating inclusion criteria and participants' demographic data (see Table 3 in the Appendix). However, numerous studies displayed significant differences in gender representation among participants, with a particularly low proportion of male participants compared to females. This imbalance presents difficulties in making meaningful comparisons.

Discussion

VADs have demonstrated significant effectiveness in treating patients with HF due to DCM. Various studies have explored the benefits of VADs in improving cardiac function, survival rates, and quality of life among these patients.

VADs have shown significant effectiveness in patients with HF due to DCM. In a study involving 800 patients with non-ischemic dilated cardiomyopathy (NICM), 460 patients (57%) experienced an improvement in ejection fraction (impEF) with a median time to improvement of 13 months [41]. Among these patients, those who maintained a persistent impEF had a lower risk of adverse outcomes, such as all-cause death, HTx, or the need for a left ventricular assist device (D/HT/LVAD), compared to those with transient impEF. The hazard ratio (HR) for D/HT/LVAD in patients with transient impEF was 2.54, indicating more than double the risk compared to patients with persistent impEF [41]. VADs played a crucial role in supporting patients with severe HF, improving survival rates, and providing a bridge to HTx. Continuous monitoring of left ventricular ejection fraction (LVEF) is essential, as a recurrent decline in LVEF was observed in approximately 40% of patients with impEF, underscoring the importance of vigilant follow-up and management in this population​ [41]. In another study involving DCM patients, the use of VADs led to substantial reductions in myocardial fibrosis and cardiomyocyte hypertrophy. Specifically, fibrosis levels in the left ventricle (LV) decreased approximately two-fold, and cardiomyocyte cross-sectional area reduced significantly in both the LV and right ventricle (RV) compared to those without VAD support [45].

VADs facilitated reverse ventricular remodeling, normalizing the expression of key genes involved in immune response, oxygen homeostasis, and extracellular matrix remodeling. For effective management, continuous monitoring and tailored therapy adjustments are crucial to optimize patient outcomes and mitigate complications associated with VAD implantation​ [45]. In a study of 102 patients with idiopathic dilated cardiomyopathy (IDC) and an LVEF of less than 40%, those who received optimal pharmacological treatment and implantable devices, including VADs, showed substantial improvements ​[42]. Over 13 months, the average LVEF increased from 26% to 40%, and peak oxygen consumption improved from 19.5 ml/kg/min to 23.4 ml/kg/min. Additionally, the New York Heart Association (NYHA) functional class significantly improved, with the majority of patients moving from class III/IV to class I/II. VADs played a crucial role as a bridge to transplantation, improving survival rates and overall cardiac function. Continuous monitoring and adherence to current HF treatment guidelines are essential to optimize patient outcomes​ [42].

A study found no significant difference in long-term survival between patients with ischemic cardiomyopathy (ICM) and those with DCM following VAD implantation, as indicated by Kaplan-Meier survival estimates (p = 0.105)​ [40]. Notably, patients with ICM tended to undergo additional cardiac procedures during VAD surgery more frequently than those with DCM (36% vs. 12%; p = 0.052) [40]. VADs play a crucial role in enhancing hemodynamic stability and organ perfusion, leading to improved clinical management and outcomes for HF patients [40]. 

VADs play a crucial role in managing HF due to DCM by providing effective mechanical circulatory support and bridging patients to HTx. In a study of 22 patients with 24 HeartWare Ventricular Assist Devices (HVAD) implants, 13 patients had DCM, including those with anthracycline-induced cardiotoxicity [35]. The mean support time was 357 days, with all patients successfully bridged to transplant. However, thrombus formation on the inflow cannula was a significant complication, observed in 96% of devices, and associated with thromboembolic events in 41% of patients, including a 27% stroke rate. These findings underscore the effectiveness of VADs in improving survival and transplant outcomes while highlighting the need for careful monitoring and management of thrombus-related complications through anticoagulation and device design improvements​ [35].

VADs have proven to be very effective, for example, in a study involving 90 patients with DCM who underwent VAD implantation, the one-year survival rate was 78.2%, with an overall survival rate of 61% by the time of data collection. Patients showed significant improvement in LVEF, with a mean preoperative LVEF of 13.8% increasing substantially post-implantation​ [21]. VADs played a critical role in reducing symptoms and increasing exercise capacity, allowing patients to engage in daily activities. Additionally, these devices provided essential circulatory support as a bridge to transplantation or as long-term DT for those not eligible for transplant. Despite the benefits, complications such as anemia (observed in 91% of patients) and kidney failure (48% experiencing transient deterioration) were noted, necessitating careful monitoring and management to optimize patient outcomes​ [21]. Another study found that the overall prevalence of ventricular arrhythmias (VAs) post-implantation was 49%, with 46% experiencing monomorphic ventricular tachycardia and 11% experiencing ventricular fibrillation (VF). Pre-existing VAs and atrial fibrillation (AF) were strong predictors of post-LVAD VAs, with pre-LVAD VAs having an HR of 5.36 and AF having an HR of 3.1 [27].

In a study of 12 patients who underwent LVAD explantation, the median LVEF improved from 20% at the time of LVAD implantation to 54% before explantation, with a statistically significant p-value of less than 0.001 [24]. The median duration of LVAD support was 10 months, during which patients showed notable cardiac recovery, enabling successful explantation without perioperative complications. Post-explantation follow-up revealed sustained myocardial recovery, with stable LV function and dimensions observed over a median follow-up period of 43 months. This study underscores the importance of a standardized weaning protocol and thorough surgical strategy for optimal long-term outcomes in VAD therapy​ [24]. Another study of 60 patients with stage D HF receiving either pulsatile flow (PF) or continuous flow (CF) VADs found that left ventricular reverse remodeling (LVRR) was achieved in 26.7% of patients, defined by an LVEF of 35% or greater within six months of support. Specifically, PF VAD usage and insufficient preoperative beta-blocker treatment were identified as independent predictors for achieving LVRR. Among those achieving LVRR, 10% had their VADs explanted within six months, all with an LVEF of at least 35% before explanation [39]. The study highlighted that patients who achieved LVRR had better clinical outcomes, including reduced aortic valve insufficiency and lower plasma B-type natriuretic peptide levels. VAD support contributed to reduced intracardiac pressure and volume, improved myocardial perfusion, and decreased cardiomyocyte hypertrophy, facilitating myocardial recovery and the potential for VAD explantation ​[39].

VADs significantly improve clinical outcomes in patients with HF due to DCM. In a study of 120 patients, including 96 with DCM and 24 with hypertrophic cardiomyopathy with left ventricular systolic dysfunction (HCM-LVSD), VAD implantation resulted in comparable overall survival rates between the groups. For DCM patients, the one-year survival rate was 94.3%, and the five-year survival rate was 88.8%, while for HCM-LVSD patients, these rates were 95.7% and 64.6%, respectively [38]. Post-implantation, brain natriuretic peptide (BNP) levels decreased significantly in both groups, from a median of 538 pg/mL to 88 pg/mL in DCM patients and from 533 pg/mL to 256 pg/mL in HCM-LVSD patients at three months post-implantation (p < 0.001) [38]. The study highlighted that despite higher postoperative BNP levels indicating subclinical right ventricular (RV) dysfunction, VADs effectively compensated for hemodynamic deficiencies, facilitating improved patient outcomes in both groups [38].

In a study involving 14 patients, VAD support for an average of 2.5 ± 1.2 years resulted in notable reductions in LV end-diastolic and end-systolic dimensions, with statistically significant decreases in these measurements post-support (p < 0.05). LV ejection fraction also significantly increased after VAD support (p < 0.05). Additionally, the serum level of BNP, a marker for HF, was significantly reduced (p < 0.01) [34]. Histologically, cardiac myocytes were smaller, and the percentage of interstitial fibrosis in the LV tissue decreased significantly after VAD support (p < 0.01) [34]. VADs facilitated these improvements by reducing LV wall stress and improving regional blood flow, which helped reverse pathological remodeling and improve overall cardiac function​ [34].

In a multicenter study involving 150 patients with end-stage HF, the use of the Jarvik 2000 VAD showed significant positive outcomes [23]. The primary endpoint, defined as successful transplantation or being listed for transplantation at 180 days, was achieved in 67.3% of the total cohort, surpassing the pre-specified threshold of 65% (95% CI: 59.5%-74.3%; p = 0.006). Notably, in the subgroup with cone bearings, the success rate was even higher at 91% (95% CI: 72%-97.5%; p = 0.001). The device played a crucial role by reducing hemolysis and end-organ dysfunction compared to its predecessors with pin bearings. Overall, VADs significantly improved the functional and quality of life scores for these patients, demonstrating their effectiveness as a bridge to transplantation​ [23]. The use of VADs demonstrated notable efficacy, with an overall survival rate of 62.8% and a median survival time of 2.78 years. Patients presented with a median LV ejection fraction of 14.5% and an LV dimension of 7.55 cm [20]​. Post-implantation, the median intensive care unit stay was six days, and the median time to discharge was 31.5 days. The primary complications included RV failure, with in-hospital mortality attributed to RV failure at 10%. Device-related issues such as clotting disorders occurred in 9% of patients. The data underscore the importance of appropriate patient selection and timing for VAD implantation to optimize outcomes ​[20].

In a cohort of 24 patients with an average age of 58.9 years, the implementation of the HeartMate 3 (HM3) VAD resulted in notable clinical improvements ​[19]. Specifically, the left ventricular end-diastolic diameter (LVEDD) decreased from 5.2 cm at baseline to 4.5 cm following increases in VAD speed, reflecting a reduction of -1.17 cm/krpm. Additionally, the LVEF improved significantly from pre-implantation levels, and the plasma BNP levels, a marker of HF, were significantly lower post-implantation. The use of VADs also stabilized hemodynamic parameters, with mean arterial blood pressure maintained within optimal ranges during different VAD speeds. These devices facilitated reverse remodeling of the heart, evidenced by smaller LV dimensions, and improved myocardial function, thereby supporting their role in the management of advanced HF in this patient population [19].

The MOMENTUM 3 study highlighted that patients receiving the HM3 device as DT achieved a two-year event-free survival rate of 73.2%, compared to 58.7% for those with the HeartMate 2. The two-year overall survival was 76.7% for HM3 patients versus 82.7% for those eligible for transplantation. Additionally, HM3 patients had an 88.1% stroke-free survival rate and a 97.5% pump thrombosis-free survival rate. These devices played a crucial role by significantly reducing adverse events, improving hemodynamic parameters, and enhancing functional status, with 80% of patients reaching NYHA class I or II two years post-implantation​ [43]. A large number of patients underwent LVAD implantation, and 44% had DCM. Post-implantation, there was a notable reduction in HF symptoms and overall prognosis improvement. The mean age of patients was 54.7 years, with 85% being male ​[18]. Specifically, LVADs significantly decreased the amplitude of the R wave in several leads (e.g., lead I from 0.3 ± 0.33 mV to 0.19 ± 0.22 mV, p < 0.0001) and altered the R ratio, particularly in leads I, II, and a VF, which are critical for ECG-based screening of subcutaneous ICD therapy. This reduction in R wave amplitude and changes in the R ratio underscores the role of LVADs in modifying cardiac electrical activity, potentially impacting continuous ICD therapy eligibility ​[18].

VADs significantly improve survival and cardiac function in patients with HF due to DCM. In a study involving 50 patients with NIDCM, four patients showed substantial cardiac functional recovery post-LVAD implantation, evidenced by improved LVEF, reductions in LV size, and decreased serum BNP levels. Specifically, LVEF improvements were associated with higher numbers of CD68-positive macrophages and CD3-positive T cells in the myocardium, indicating the role of immunocompetent cells in myocardial recovery [33]. Event-free survival rates at one and five years were 96% and 89%, respectively, highlighting the long-term efficacy of LVADs in reducing hospital readmissions and improving patient outcomes ​[33].

The use of large Impella systems, such as Impella 5.0 or 5.5, has shown promising results in improving hemodynamics and survival rates. In a study of 50 patients treated with Impella 5+, the 30-day survival rate was 56%, although in-hospital mortality remained high at 50%. Notably, patients with DCM had a significantly higher mortality rate (p = 0.02, OR 15.8) compared to those with ICM, who showed better outcomes (p = 0.03, OR 0.24)​ [31]. The Impella devices provided effective LV unloading, reduced myocardial oxygen consumption, and improved CO, which stabilized the patients' conditions and facilitated recovery [31]. Additionally, the combination of Impella with veno-arterial extracorporeal membrane oxygenation and temporary right ventricular assist devices (RVAD) further enhanced the weaning process from mechanical support, contributing to better short-term outcomes​ [31]. In a study involving 26 patients, 70% of whom had idiopathic DCM, VADs were explanted after a median support duration of 317 days. Post-explant, the mean LVEF at one year was 44.25% ± 8.44%, with a Kaplan-Meier estimated survival of 88% at one year and 77% at six years [26]. The role of VADs was pivotal in myocardial recovery, providing LV unloading that facilitated improved cardiac function and survival​ [26]. This effectiveness was underscored by the standardized weaning protocol and minimally invasive explant techniques employed, which minimized periprocedural complications and supported long-term survival​ ​[26].

In a study of 92 patients with cardiogenic shock (CS), 26 patients with decompensated DCM were included. The 30-day survival rate was 63%, with survival significantly associated with a higher baseline LVEF, lower serum lactate levels, and shorter duration of invasive ventilation. Patients with higher LVEF (21.8% vs. 15.1%, p < 0.001) and lower serum lactate (1.45 mmol/L vs. 2.8 mmol/L, p = 0.012) had better outcomes [32]. Prolonged invasive ventilation (>24 hours) was linked to a lower survival rate (64.7% vs. 13.8%, p < 0.001). The Impella devices played a crucial role in mechanically unloading the LV, reducing myocardial oxygen demand, and improving myocardial perfusion, which facilitated hemodynamic stabilization and recovery​ [32]. The median support time for patients using VADs was 410 days, with a significant portion achieving substantial recovery. Notably, 88% of patients remained free from death, LVAD reimplantation, HTx, and significant HF relapse at 24 months post-explantation ​[25].

VADs play a crucial role in stabilizing hemodynamics, reducing myocardial oxygen demand, and improving LV function, as evidenced by the increase in LVEF from 17% to 19% and the reduction in LVEDD from 71.3 mm to 65.6 mm​ [25]. This underscores the potential of VADs not only as a bridge to recovery but also in providing long-term cardiac support and enhancing survival rates for these patients​ [25]. In a study of 75 patients, those who achieved optimal mechanical unloading (defined as a reduction in LVEDD of ≥15% at 6 months) showed significant improvements in various cardiac parameters [29]. The optimally unloaded group had a mean fractional shortening of 15% ± 7% compared to 10% ± 7% in the poorly unloaded group (p = 0.007). Additionally, rates of moderate or severe mitral regurgitation were lower in the optimally unloaded group (10% vs. 33%, p = 0.02). Pulmonary capillary wedge pressure (PCWP) was also significantly lower (9 ± 4 mmHg vs. 16 ± 7 mmHg, p = 0.02) [29].

In a study involving 13 patients with DCM who underwent biventricular support using HVAD pumps, the results indicated a median support duration of 269 days, with a 30-day survival rate of 100% and a one-year survival rate of 62%. The RVAD was implanted in the RV-free wall in six patients and in the right atrial (RA) free wall in seven patients. The RA group experienced fewer complications related to pump thrombosis (14% in the RA group vs. 50% in the RV group) and bleeding (0% in the RA group vs. 50% in the RV group). However, the overall survival rate at two years was 54%, with 38% of patients successfully bridged to transplantation​ [30].

Significant improvements can be picked up in several key measures after VADs implantation. A study found that LVEDD decreased from 6.49 cm to 4.94 cm (p < 0.0001), LVEF increased from 20.3% to 46.9% (p < 0.0001), PCWP decreased from 25.9 mm Hg to 11.5 mm Hg (p < 0.0001), and CO increased from 3.73 L/min to 6.41 L/min (p < 0.0001) [46]. These devices played a critical role in unloading the LV, thereby reducing myocardial oxygen demand and promoting myocardial recovery, leading to improved cardiac function and allowing for the potential of device explantation and return to medical management [46].

In a study analyzing 677 patients with familial dilated cardiomyopathy (FDCM), those who received LVAD exhibited significantly improved survival rates compared to those with NICM and ICM. The study found that FDCM patients had a one-year post-transplant survival rate of 91%, three-year survival of 88%, and five-year survival of 80%, which were similar to NICM (91%, 84%, 79%) and superior to ICM (89%, 82%, 75%) (p = 0.008). Furthermore, FDCM patients were less likely to die or be delisted due to clinical deterioration compared to NICM (HR: 0.62, 95%CI: 0.47-0.81) and ICM (HR: 0.5, 95%CI: 0.38-0.66), and more likely to be transplanted compared to both NICM (HR: 1.25, 95%CI: 1.14-1.37) and ICM (HR: 1.18, 95%CI: 1.08-1.3). VADs play a crucial role in stabilizing patients by reducing myocardial workload and improving hemodynamics, thereby facilitating myocardial recovery and improving the likelihood of successful transplantation [28].

VADs significantly improve survival rates and clinical outcomes for patients with HF due to DCM. In a prospective study of 772 patients admitted for CS, 78 with IDCM had higher rates of death or cardiovascular rehospitalizations compared to non-IDCM patients, with an adjusted OR of 4.77 (95% CI 1.13 to 20.1, p = 0.03). Patients under 65 years in the IDCM group had a significantly higher need for HTx or VAD, with an adjusted OR of 2.68 (95% CI 1.21 to 5.91, p = 0.02). VADs provided mechanical circulatory support, stabilized hemodynamics, and enabled myocardial recovery, improving long-term outcomes [37]. Another study with 125 patients who underwent LVAD implantation, including 44 with DCM, reported a one-year survival rate of 78.2% and overall survival of 61%, with a median follow-up of 30 months. Key complications included anemia in 91% and kidney failure in 44% pre-implantation and 48% post-implantation. LVADs reduced symptoms, stabilized hemodynamics, and improved quality of life, allowing significant functional recovery and successful HTx in many patients [21].

In a study involving 347 patients with DCM who required either an LVAD or heart transplant, 26.2% had pathogenic or likely pathogenic (P/LP) genetic variants, compared to 15.9% of patients with an ICD and 15.0% with neither intervention. Patients with advanced DCM were more than twice as likely to carry a P/LP variant than those with less severe disease (OR 2.3, 95% CI 1.5-3.6). The role of VADs was crucial in providing mechanical circulatory support, reducing myocardial workload, and stabilizing hemodynamics, thereby facilitating myocardial recovery and improving the likelihood of successful HTx or long-term survival ​[22]. In a study validating the VT-LVAD score, which predicts the occurrence of late VAs in patients post-LVAD implantation, 357 patients were analyzed [17]. The study found that the VT-LVAD score accurately predicted late VAs in high-risk groups, with 130 patients experiencing late VAs after a median follow-up of 25 months. The VT-LVAD score, which includes factors such as VAs prior to LVAD, absence of ACE inhibitors, HF duration over 12 months, early VAs post-LVAD, AF, and idiopathic DCM, was a strong independent predictor (p < 0.001, OR: 4.8) [17].

VADs significantly improve survival rates and clinical outcomes in patients with HF due to DCM. In a retrospective study involving 79 patients with end-stage HF, including 27 patients with DCM, the mean duration of CF-LVAD support was 604 days. The study reported a one-year survival rate of 81%, with 70% of patients surviving for two years and 61% for three years. Notably, the mean length of hospital stay was 61 days, with a mean intensive care unit stay of 14 days. The use of CF-LVADs played a crucial role in stabilizing hemodynamics, reducing HF symptoms, and improving overall quality of life, thereby facilitating significant functional recovery and allowing many patients to successfully bridge to HTx or achieve long-term support [44].

In a study of 60 patients, LVAD support for six months led to 26.7% of patients achieving an LVEF of ≥35%, indicating substantial improvement. Notably, the use of PF LVADs resulted in greater enhancements in LVEF and reduced LVEDD compared to CF-LVADs. Patients with lower preoperative beta-blocker treatment were more likely to achieve reverse remodeling, underscoring the importance of preoperative medical therapy in predicting positive outcomes [16]. Similarly, the Impella 5.0, an axial-flow percutaneous VAD used in patients with cardiogenic shock, is typically recommended for up to 10 days. However, due to hemodynamic instability, its use can be extended, leading to potential device malfunctions requiring replacement [16]. Overall, both PF LVADs and the Impella 5.0 highlight the crucial role of VADs in structural and functional cardiac recovery, while emphasizing the importance of careful management to mitigate complications and optimize patient outcomes.

## Conclusions

VADs have proven highly effective in the management of HF due to DCM. The studies reviewed demonstrate that VADs significantly improve LVEF, reduce myocardial fibrosis, and facilitate reverse ventricular remodeling. They play a crucial role in stabilizing hemodynamics, reducing symptoms, and enhancing survival rates. VADs also serve as a bridge to HTx, with many patients showing substantial improvements in cardiac function and quality of life. However, complications such as thrombus formation, anemia, and kidney failure necessitate careful monitoring and management. Continuous advancements in device design and patient management protocols are essential to further optimizing outcomes. Overall, VADs are invaluable in the treatment of advanced HF, providing both immediate hemodynamic support and long-term therapeutic benefits.

## Appendices

JBI critical appraisal tool

**Table 3:** Results for the analysis of study quality or bias using the JBI Critical Appraisal Tools for the included studies Y: yes; N: no; NC: not clear; JBI: Joanna Briggs Institute

Checklist question	Oishi et al. [16]	Darma et al. [17]	Zormpas et al. [18]	Schlöglhofer et al. [19]	Wasilewski et al. [20]	Wasilewski et al. [21]	Hofmeyer et al. [22]	Selzman et al. [23]	Gyoten et al. [24]	Antonides et al. [25]	Vela et al. [26]	Efimova et al. [27]	Khayata et al. [28]	William et al. [29]	Shehab et al. [30]	Sugimura et al. [31]	Panoulas et al. [32]	Koga-Ikuta et al. [33]	Ito et al. [34]	Glass et al. [35]	Sammani et al. [36]	Cherbi et al. [37]	Yagi et al. [38]	Imamura et al. [39]	Ivanov et al. [40]	Manca et al. [41]	Broch et al. [42]	Kuśmierczyk et al. [43]	Biełka et al. [44]	Parikh et al. [45]	Frazier et al. [46]
Were there clear criteria for inclusion in the case series?	Y	Y	Y	Y	Y	Y	Y	Y	Y	Y	Y	Y	Y	Y	Y	Y	Y	Y	Y	Y	N	Y	Y	Y	Y	Y	Y	Y	Y	Y	Y
Was the condition measured in a standard, reliable way for all participants included in the case series?	Y	Y	Y	Y	Y	N	N	Y	Y	N/C	Y	Y	Y	N/C	Y	Y	Y	Y	Y	Y	N	Y	Y	Y	Y	Y	Y	Y	Y	Y	Y
Were valid methods used for the identification of the condition for all participants included in the case series?	Y	Y	Y	Y	Y	Y	N/C	Y	Y	Y	Y	Y	Y	Y	Y	N/C	N/C	Y	Y	Y	Y	Y	Y	Y	Y	Y	Y	Y	Y	Y	Y
Did the case series have the consecutive inclusion of participants?	NC	Y	Y	Y	Y	Y	Y	Y	Y	Y	Y	Y	Y	Y	Y	Y	Y	Y	Y	Y	Y	Y	Y	Y	Y	Y	Y	Y	Y	Y	Y
Did the case series have a complete inclusion of participants?	Y	Y	Y	Y	Y	Y	Y	Y	Y	Y	Y	Y	Y	Y	Y	Y	Y	N/C	Y	Y	Y	N/C	N/C	Y	Y	Y	Y	N/C	Y	Y	Y
Was there clear reporting of the demographics of the participants in the study?	Y	Y	Y	Y	Y	Y	Y	Y	Y	Y	Y	Y	Y	Y	Y	Y	Y	N	Y	Y	Y	Y	Y	Y	Y	Y	Y	Y	Y	Y	Y
Were the outcomes or follow-up results of cases clearly reported?	Y	Y	Y	Y	Y	Y	Y	Y	Y	Y	Y	Y	Y	Y	Y	Y	Y	N/C	Y	Y	Y	Y	Y	Y	Y	Y	Y	Y	Y	Y	N/C
Was there clear reporting of the presenting sites’ or clinics’ demographic information?	Y	Y	Y	Y	Y	Y	Y	Y	Y	Y	Y	Y	Y	Y	N/C	N/C	Y	Y	Y	Y	N/C	Y	Y	Y	Y	Y	Y	Y	Y	Y	Y
Was the statistical analysis adequate?	Y	Y		Y	Y	Y	Y	Y	Y	Y	Y	Y	Y	Y	Y	Y	Y	Y	Y	Y	Y	Y	Y	Y	Y	Y	Y	Y	Y	Y	Y
